# Exploring the roles of FGF/MAPK and cVG1/GDF signalling on mesendoderm induction and convergent extension during chick primitive streak formation

**DOI:** 10.1007/s00427-022-00696-1

**Published:** 2022-09-23

**Authors:** Hyung Chul Lee, Nidia M. M. Oliveira, Claudio D. Stern

**Affiliations:** Department of Cell and Developmental Biology, University College London, Gower Street, London, WC1E 6BT UK

**Keywords:** Gastrulation, Embryonic polarity, Planar cell polarity, Primitive streak elongation, Epithelial cell intercalation

## Abstract

During primitive streak formation in the chick embryo, cells undergo mesendoderm specification and convergent extension at the same time and in the same cells. Previous work has implicated cVG1 (*GDF3*) as a key factor for induction of primitive streak identity and positioning the primitive streak, whereas FGF signalling was implicated in regulating cell intercalation via regulation of components of the WNT-planar cell polarity (PCP) pathway. FGF has also been reported to be able to induce a primitive streak (but lacking the most axial derivatives such as notochord/prechordal mesendoderm). These signals emanate from different cell populations in the embryo, so how do they interact to ensure that the same cells undergo both cell intercalation and acquire primitive streak identity? Here we begin to address this question by examining in more detail the ability of the two classes of signals in regulating the two developmental events. Using misexpression of inducers and/or exposure to inhibitors and in situ hybridisation, we study how these two signals regulate expression of Brachyury (*TBXT*) and *PRICKLE1* as markers for the primitive streak and the PCP, respectively. We find that both signals can induce both properties, but while FGF seems to be required for induction of the streak by cVG1, it is not necessary for induction of *PRICKLE1*. The results are consistent with cVG1 being a common regulator for both primitive streak identity and the initiation of convergent extension that leads to streak elongation.

## Introduction

Formation of the primitive streak in early amniote embryos involves the acquisition of several distinct properties by the same cells at about the same time. These properties include expressing specific markers of primitive streak “identity” (such as *Brachyury/TBXT*, *SNAI2*, *TBX6*), epithelial-to-mesenchymal transition (EMT) leading to cell ingression and epithelial cell intercalation (convergent extension), leading to elongation of the primitive streak along the midline. In the chick embryo, primitive streak formation can be initiated from any position around the circumference of the disc-shaped embryonic epiblast. Therefore, some key questions remaining to be answered include the following: what mechanisms are responsible for the same cell population acquiring all of these properties, what signals regulate each of the properties and how are they integrated by the responding cells?

Previous studies in the chick embryo have shown that cVG1 (annotated *GDF3* in the chicken genome, although synteny suggests that this gene should more appropriately be considered as the orthologue of *GDF1*), a member of transforming growth factor (TGF)-beta superfamily, is expressed in the posterior marginal zone and is an important factor for positioning the primitive streak (Bertocchini and Stern 2012; Shah et al. 1997; Skromne and Stern 2002). Grafting a cell pellet expressing cVG1 in non-posterior regions of pre-primitive-streak stage embryos can induce primitive streak formation with hierarchical gene expression (Shah et al. 1997; Skromne and Stern 2002), and inhibition of cVG1 using morpholino oligonucleotides impairs primitive streak formation (Bertocchini and Stern 2012). Fibroblast growth factor (FGF) signalling has been shown to cooperate with cVG1 to induce the primitive streak (Bertocchini et al. 2004).

Other studies showed that primitive streak elongation involves medio-lateral cell intercalation (convergent-extension) of future primitive streak cells (Voiculescu et al. 2007, 2014), and that this involves the WNT-planar cell polarity (PCP) pathway (Voiculescu et al. 2007) acting through myosin-II (Rozbicki et al. 2015). Three PCP-pathway components, Flamingo (CELSR), PRICKLE1 and VANGL2 were shown to play a role in medio-lateral intercalation in the epiblast based on their overlapping expression and knockdown experiments with morpholinos (Voiculescu et al. 2007). This was confirmed with experiments involving misexpression of Dishevelled lacking the DEP domain, which acts as a dominant-negative (Rothbacher et al. 2000): this generates a broad and thickened *TBXT*-expressing domain without a morphological “streak” (Voiculescu et al. 2007), revealing that specification of the primitive steak identity (mesendoderm specification) and the convergent extension of the epiblast cells can be separated.

FGF signalling from the hypoblast (a deep layer under the epiblast, which eventually will contribute to the yolk sac stalk (Stern and Downs 2012)) regulates cell movements by inducing the expression of the WNT/PCP pathway genes in cells that will later form the primitive steak (Foley et al. 2000; Voiculescu et al. 2007). However, pre-primitive-streak stage embryos from which the hypoblast layer has been removed can still generate the primitive streak (and even multiple primitive streaks), which elongate(s) appropriately (Bertocchini and Stern 2002). This result raises the question whether FGF signalling derived from the hypoblast is necessary for the induction of the WNT/PCP pathway, and if not, what other signals are involved.

Here we explore the relative roles of FGF and cVG1 in regulating the specification of primitive streak identity and PCP components involved in cell intercalation during primitive streak formation. Our results suggest that FGF/MAPK signalling is required for cVG1 to induce *TBXT*; FGF/MAPK can also induce the PCP component *PRICKLE1* but it is not required for the induction of *PRICKLE1* by cVG1.

## Materials and methods

### Embryo harvest and whole mount in situ hybridisation

Fertilised White Leghorn hens’ eggs were obtained from Henry Stewart Farm, UK, and incubated at 38 °C until appropriate stages of embryos were obtained. Embryos were harvested in Pannett-Compton saline (Pannett and Compton 1924), then cultured by the modified New culture method (New 1955; Stern and Ireland 1981). In experiments involving grafts of cell pellets or microbeads, the cultured embryos were fixed in 4% paraformaldehyde in phosphate buffered saline at 4 °C overnight, and then whole mount in situ hybridisation was conducted as previously described (Stern 1998; Streit and Stern 2001). The probes we used were *FGF8* (Streit and Stern 1999), *TBXT* (Kispert et al. 1995) and *PRICKLE1* (Voiculescu et al. 2007). Some embryos were embedded in paraffin wax and sectioned using a Zeiss Microm microtome (HM315) at 10 μm thickness as previously described (Streit and Stern 2001). Whole embryos and sections were observed under an Olympus SZH10 stereo microscope or an Olympus Vanox-T optical microscope, respectively, and then imaged with a QImaging Retiga 2000R camera.

### Misexpression of proteins with transfected cell pellets

Misexpression of proteins was conducted as previously described (Lee et al. 2022). Briefly, HEK293T cells were cultured in 10% foetal bovine serum in Dulbecco’s Modified Eagle Medium and transfected using PEI as reported previously (Papanayotou et al. 2013). Expression plasmids were DMVg1 for cVG1 expression (Shah et al. 1997) and pMT23 (murine BMP4 (Dickinson et al. 1990)) for BMP4 expression. Then, transfected cells were cultured for 36–48 h at 37 °C in a hanging drop to make a cell pellet ranging in size from 500 to 1000 cells.

### Delivery of proteins and inhibitor via microbeads

Fifty micrograms per millilitre of mouse FGF8b (Sigma-Aldrich, F6926) in 0.1% bovine serum albumin in phosphate-buffered saline was delivered using heparin-acrylic beads (Sigma-Aldrich, H5236), while 250 μM SU5402 (Calbiochem, 572,630), 1 mM U0126 (Calbiochem, 662,005) and 1 mM U0124 (Calbiochem, 662,006) in DMSO were loaded onto AG1X2-formate beads (Streit et al. 2000). In each case, the beads were incubated overnight at 4 °C in the protein or chemical at the concentrations stated above. Beads were washed in Pannett-Compton saline just before use. Embryos were fixed at 3 h, 6 h or 9 h after the graft or after overnight incubation.

## Results and discussion

### Expression of FGF8, TBXT and PRICKLE1 at the time of primitive streak formation

During its formation, cells of the primitive streak execute at least two cellular events at the same time, primitive-streak-cell specification and convergent extension. It has previously been shown that cVG1, WNT and FGF signals collaborate to specify primitive-streak cells (Bertocchini et al. 2004; Skromne and Stern 2001, 2002), while WNT/PCP pathway, as a downstream target of FGF, is responsible for driving convergent extension via epiblast cell intercalation (Voiculescu et al. 2007, 2014), executed via myosin-II (Rozbicki et al. 2015). It has also been shown that specification of primitive streak cells (and mesendoderm) is separable from elongation of the primitive streak by convergent extension (Voiculescu et al. 2007, 2014). However, it is less clear how these two cellular events are regulated so that they occur in the same cells. To find out what inducing factor is responsible for each downstream result, PCP pathway activation and mesoderm formation (specification of primitive-streak cells), we begun by re-investigating the spatial and temporal expression patterns of mRNA of some genes associated with these events. The expression of cVG1 has been extensively studied in our laboratory including several publications (see below); now we have also reanalysed the expression of *FGF8*, *TBXT* and *PRICKLE1*. cVG1 and FGF8 have been implicated as inducing factors for primitive streak formation (Bertocchini et al. 2004; Shah et al. 1997), while *TBXT* and *PRICKLE1* are used as makers for mesendoderm/primitive-streak cells and for the WNT/PCP pathway, respectively (Voiculescu et al. 2007). Previous studies reporting their expression patterns have mainly relied on whole-mounts and there are sometimes large gaps in the stages that have been examined; we therefore chose to follow their expression in sections (to determine the precise layers of cells that express the genes) and in a more precise time-course.

**Fig. 1:**
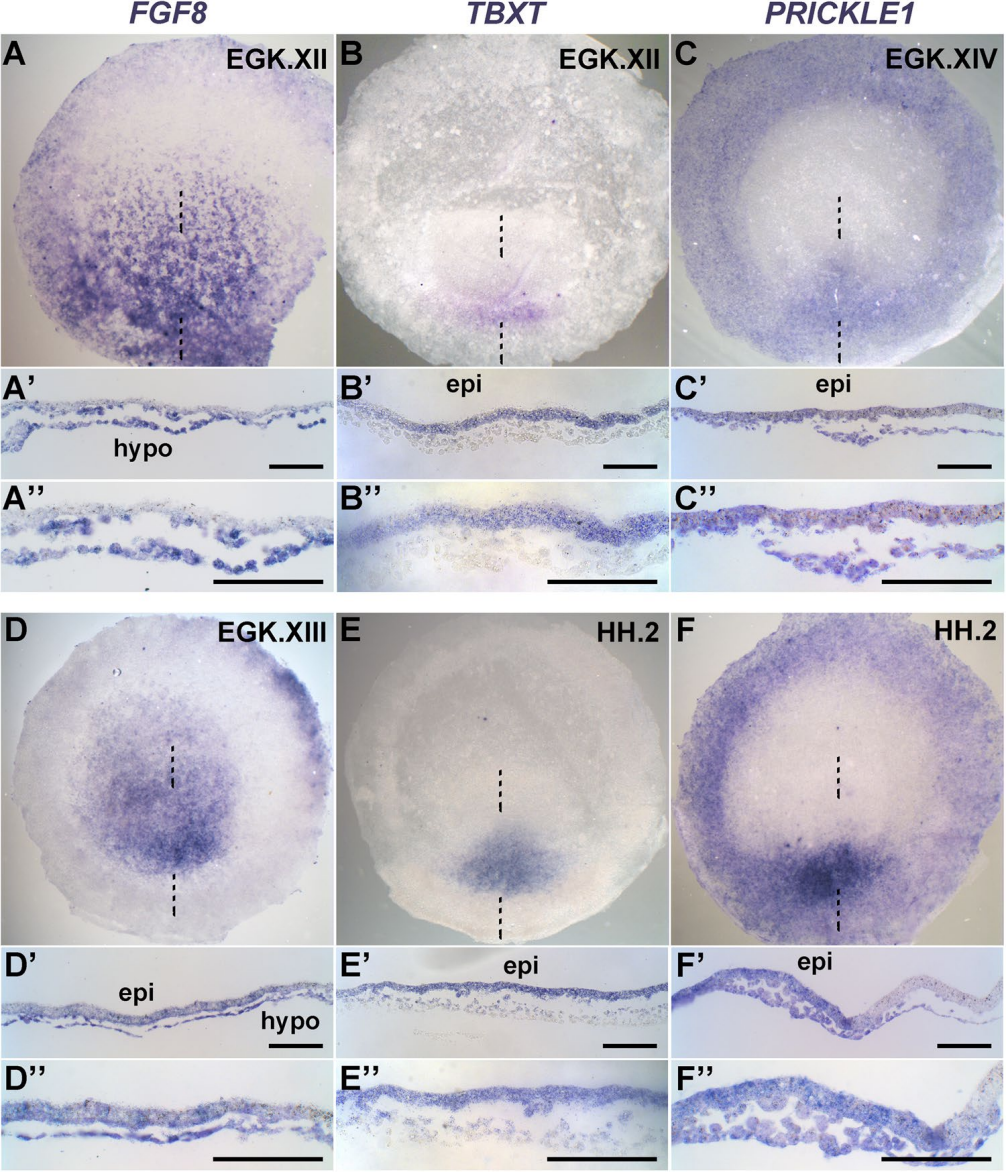
Expression patterns of *FGF8*, *TBXT* and *PRICKLE1* in the pre-primitive-streak stage embryo, revealed by in situ hybridisation. *FGF8* is detected exclusively in the hypoblast (hypo) at EGK stage XII (**A**), then in both the epiblast (epi) and the lower layers at EGK stage XIII (**D**). *TBXT* starts to be expressed in the posterior epiblast at EGK stage XII (**B**), then is also expressed in the forming mesendoderm at HH stage 2 (**E**). *PRICKLE1* is expressed both in the epiblast and the lower layers of the primitive streak at EGK stage XIV (**C** and **F**). The upper (**A**–**F**) and lower (**A**’–**F**’ and **A**’’–**F**’’, 20 × and 40 × magnification, respectively) panels correspond, respectively, to a whole mount and a sagittal section at the level of the dashed line. Scale bar, 100 µm for all sections

Expression of *cVG1* is initially (from EGK stage X) (Eyal-Giladi and Kochav 1976) restricted to the epiblast of the marginal zone, followed some hours later by expression in the newly forming mesendoderm of the primitive streak in the posterior area pellucida (Lee et al. 2020; Shah et al. 1997). *FGF8* is initially (also from EGK stage X) expressed only in the lower layers, particularly in Koller’s sickle (which defines the boundary between marginal zone and area pellucida) and the hypoblast underlying the area pellucida (Streit et al. 2000), and later (EGK XIII) both in the area pellucida epiblast and hypoblast at EGK XIII (Fig. 1A and D). A previous RNAseq analysis of pre-primitive-streak stage embryos revealed that the only member of the FGF family with significant levels of expression at these stages is *FGF8* (Lee et al. 2020). Although *FGF12* and *FGF13* are expressed at low level in the RNAseq study, these levels are too low even for detection by in situ hybridisation (Karabagli et al. 2002). These results suggest that the sickle and hypoblast are likely to be the only significant sources of FGF signals at these early stages.

Unexpectedly, we detect *TBXT* as early as stage EGK XI-XII (many hours prior to primitive streak formation) in the most posterior epiblast. As the primitive streak starts to appear (stage HH 2) (Hamburger and Hamilton 1951), *TBXT* is expressed in the forming mesendoderm of the streak including the overlying epiblast (Fig. 1B and E). *PRICKLE1* starts to be expressed from stage EGK XII-XIII in the posterior area pellucida epiblast and Koller’s sickle and intensifies in primitive streak cells from stages EGK XIV–HH 2, both in the mesendoderm and in the overlying epiblast (Fig. 1C and F). These results are consistent with either cVG1 derived from the epiblast and/or FGF8 from the hypoblast being involved in the induction of *TBXT* (marking primitive streak identity) and/or *PRICKLE1* (cells undergoing intercalation) expression in future primitive streak cells in the posterior area pellucida.

### Timing of induction of PRICKLE1 and TBXT by cVG1 and FGF

**Fig. 2:**
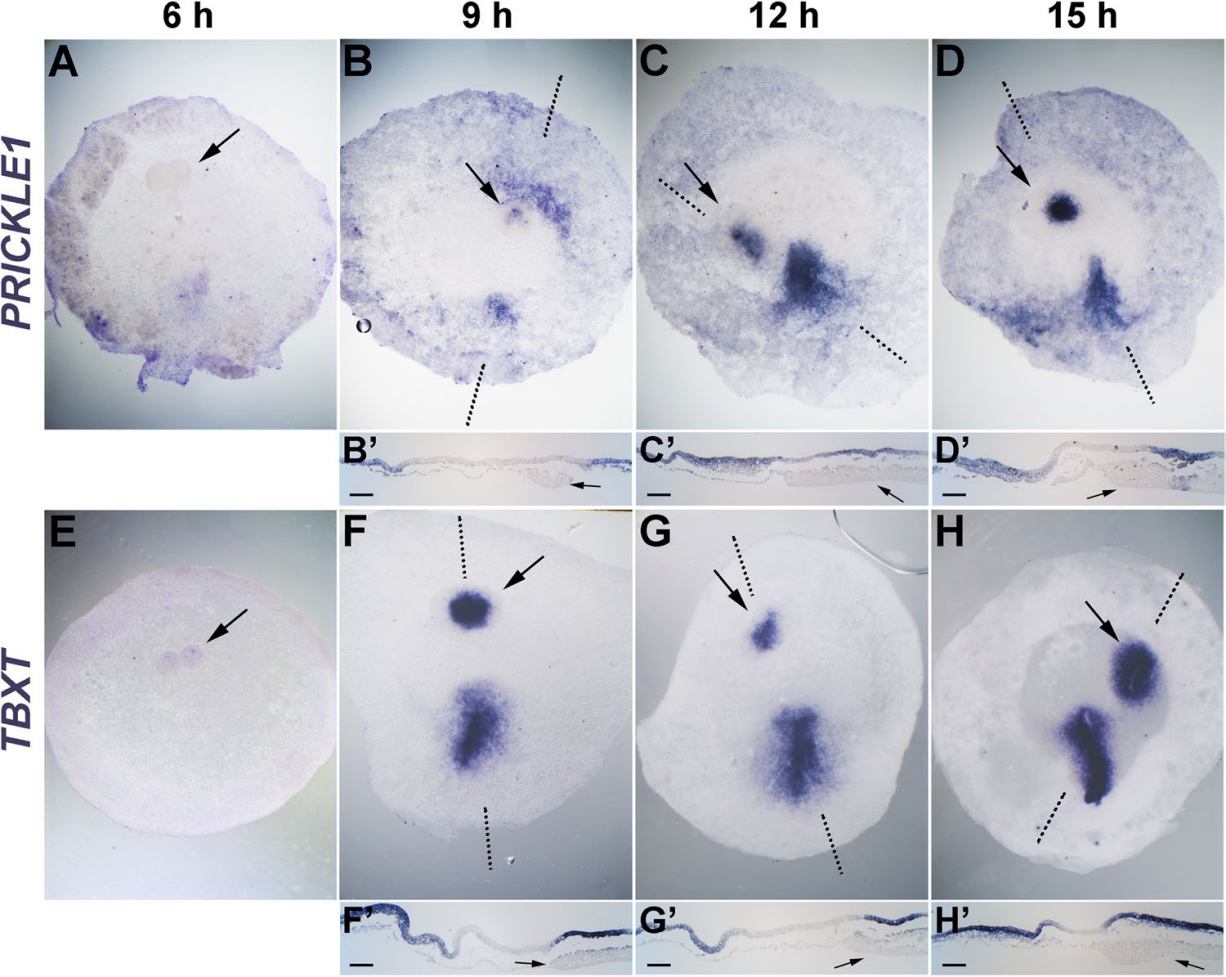
cVG1 misexpression induces *PRICKLE1* and *TBXT* expression. Two cVG1-expressing cell pellets were grafted to the anterior region of the embryo at EGK X-XI, and the expression of *PRICKLE1* (**A**–**D**) and *TBXT* (**E**–**H**) was investigated by in situ hybridisation after 6, 9, 12 and 15 h incubation. Both genes begin to be expressed from 9 h after incubation in the epiblast above the grafted cell pellet. The upper (**A**–**H**) and lower (**B**’–**D**’ and **F**’–**H**’) panels show the whole embryo and a sagittal section at the level of the dotted lines. Arrows: location of the cVG1-expressing cell pellet. Scale bars, 100 µm for all sections. The number of embryos with ectopic expression and total number of embryos in (**A**–**H**) were 0/6, 5/6, 5/6, 4/5, 0/4, 5/5, 6/6 and 5/5

Both cVG1 (Seleiro et al. 1996; Shah et al. 1997; Skromne and Stern 2002) and FGF8 (Bertocchini et al. 2004; Bertocchini and Stern 2012) induce primitive streak formation when misexpressed anteriorly or laterally﻿. To investigate the dynamics of induction of *PRICKLE1* and *TBXT* by cVG1, cVG1-expressing cell pellets were grafted to the anterior marginal zone of pre-primitive-streak stage (EGK X-XI) embryos and the expression of both genes was examined at different time points. Both genes start to be expressed by 9 h (but not 6 h) after the graft in the epiblast near the grafted cell pellet (Fig. 2). These results suggest that misexpression of cVG1 by graft of cell pellets can induce primitive-streak identity (*TBXT*) and the PCP pathway (*PRICKLE1*) in adjacent epiblast cells, with a similar time course.

**Fig. 3:**
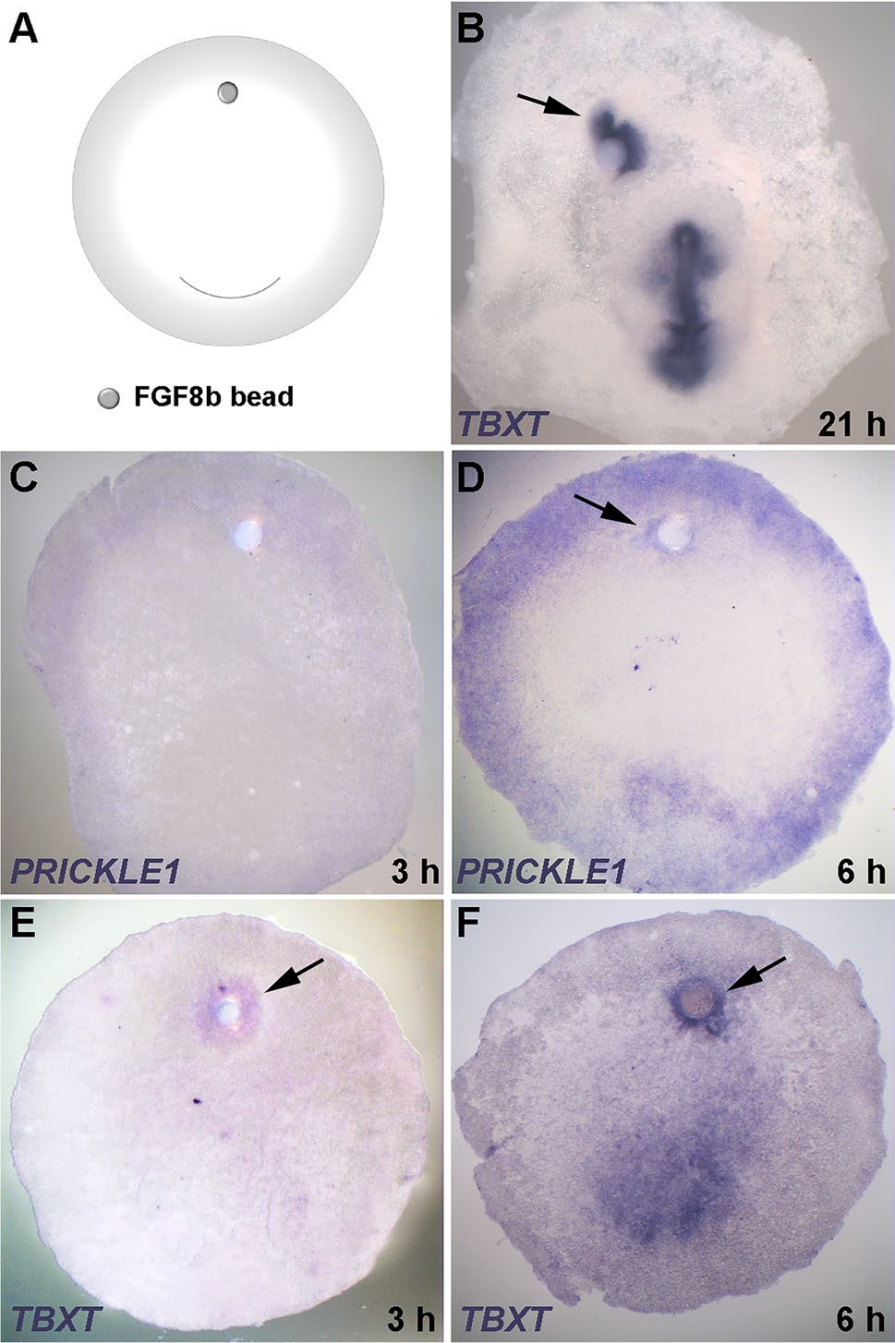
FGF8 misexpression induces *PRICKLE1* and *TBXT*. (**A**) Experimental design. An FGF8-soaked bead was grafted to the anterior region of the embryo at EGK X-XI. (**B**) After overnight culture, an ectopic primitive streak, expressing *TBXT* (arrow), was induced near the bead. (**C**–**D**) An FGF8-soaked bead induces *PRICKLE1* by 6 h after grafting (arrow, **D**). (**E**–**F**) FGF8-beads also induce weak *TBXT* expression as soon as 3 h after grafting; this later intensifies (arrows, **E** and **F**). Number of embryos with ectopic expression and total number of embryos in (**B**–**F**): 4/6, 0/5, 3/5, 4/5 and 6/6

Next, we conducted equivalent experiments with FGF8. For this, a FGF8b-soaked bead was grafted onto the anterior marginal zone of pre-primitive-streak stage embryos (Fig. 3A); this has previously been reported to induce a primitive streak (Bertocchini et al. 2004) (Fig. 3B). *PRICKLE1* was induced in adjacent cells 6 h after the graft (Fig. 3C and D), while weak expression of *TBXT* was first detectable after 3 h (Fig. 3E and F), increasing thereafter. These results show that both cVG1 and FGF8 can induce both *TBXT* (a marker of primitive streak identity) and *PRICKLE1* (marker for the PCP) in adjacent tissues. In this experiment, FGF8 appears to induce expression of both genes more quickly than does cVG1.

### FGF signalling is not required for induction and maintenance of PRICKLE1, but is required for induction of TBXT

**Fig. 4:**
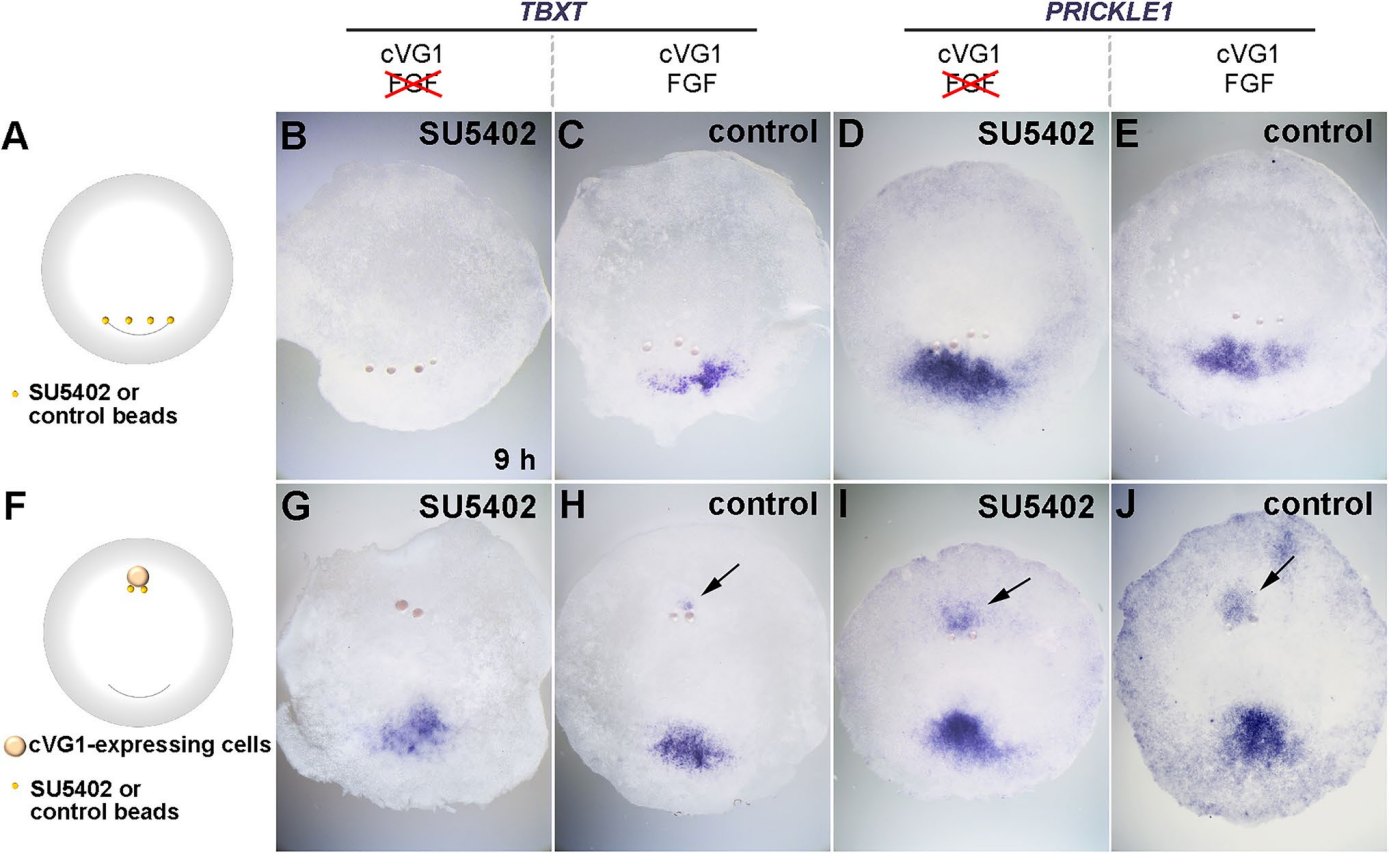
Effect of SU5402, a FGF inhibitor, on the induction of *TBXT* and *PRICKLE1*. (**A** and **F**) Experimental design. SU5402-soaked or control (DMSO) beads were grafted either into the posterior area pellucida (four beads, **A**), or into the anterior area pellucida at EGK X-XI together with a cVG1-expressing cell pellet (two beads, **F**). Then, after 9-h incubation, expression of *TBXT* or *PRICKLE1* was investigated by in situ hybridisation. (**B**–**E**) SU5402-soaked beads inhibit *TBXT* expression but not *PRICKLE1* in the posterior area pellucida (prospective primitive streak cells). (**G**–**J**) SU5402-soaked beads inhibit induction of *TBXT* but not *PRICKLE1* by a cVG1-pellet in the anterior area pellucida. Arrows, induced expression. Number of embryos with expression near the beads and total number of embryos: 0/4, 2/2, 4/4 and 3/3, respectively, for **B**–**E**, and 0/6, 4/4, 2/3 and 6/6, respectively, for **G**–**J**. Note that in some embryos, some of the beads become detached during fixation or in situ hybridisation

**Fig. 5:**
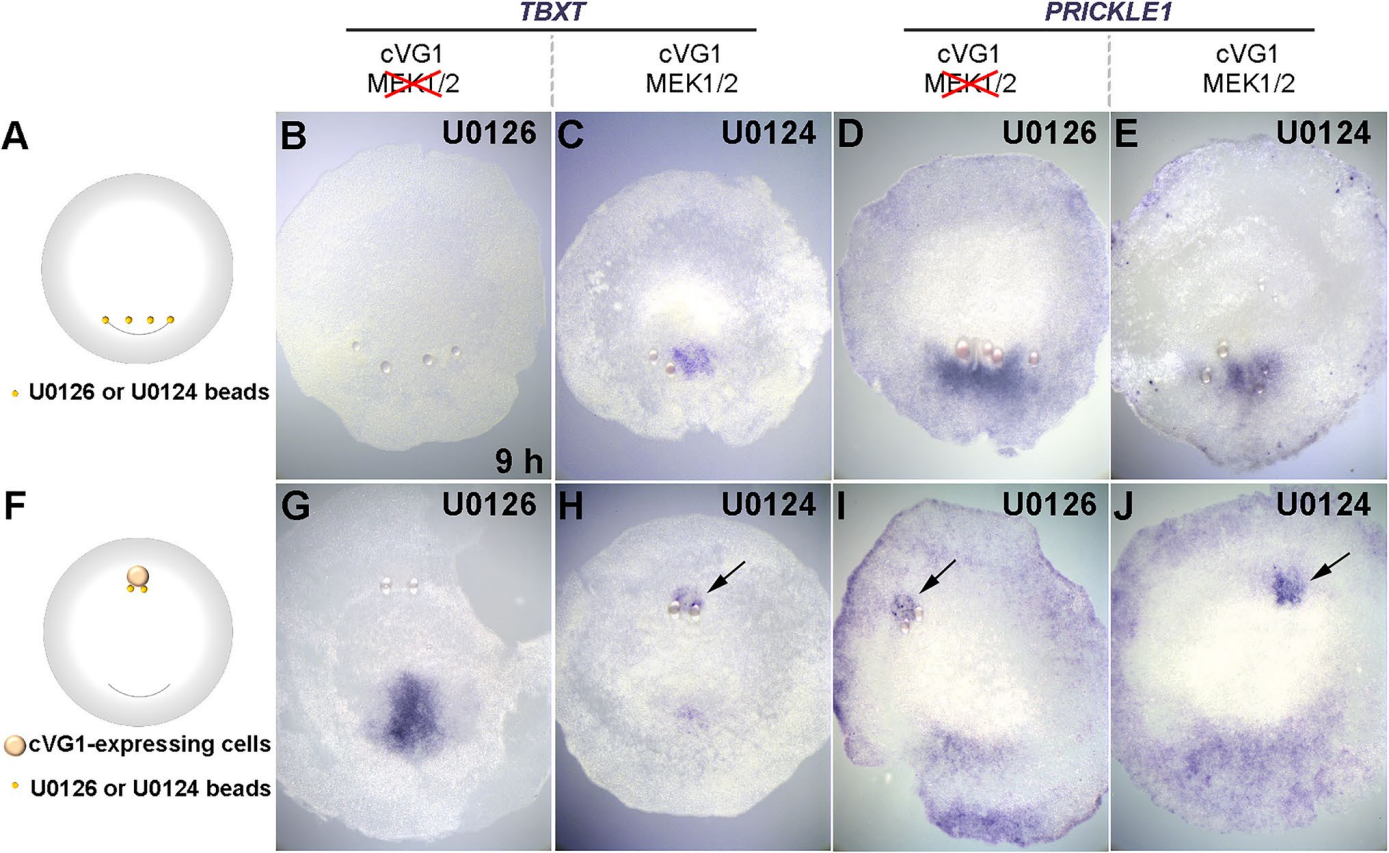
Effect of U0126, a MEK1/2 inhibitor, on the induction of *TBXT* and *PRICKLE1*. (**A** and **F**) Experimental design. U0126-soaked or control (U0124) beads were grafted into either the posterior area pellucida (four beads, **A**), or into the anterior area pellucida at EGK X-XI together with a cVG1-expressing cell pellet (two beads, **F**). Then, after 9-h incubation, expression of *TBXT* or *PRICKLE1* was investigated by in situ hybridisation. (**B**–**E**) U0126-soaked beads, but not control (U0124) beads, inhibit *TBXT* expression but not *PRICKLE1* posteriorly. (**G**–**J**) U0126-soaked beads inhibit induction of *TBXT* but not *PRICKLE1* by cVG1 anteriorly. Arrows, induced expression. Number of embryos with expression near the beads and total number of embryos: 2/12, 8/9, 11/12 and 5/8, respectively, for **B**–**E**, and 0/4, 3/4, 4/5 and 5/5, respectively, for **G**–**J**. Note that as in Fig. 4, some of the beads have become detached during processing of the embryos

Previous studies have reported that the hypoblast and one of the signalling molecules it emits, FGF8, can influence the elongation of the primitive streak by regulating the WNT/PCP pathway, and thereby cell intercalation (Voiculescu et al. 2007, 2014). However, after removal of the hypoblast layer, embryos can still generate primitive streaks that undergo elongation (Bertocchini and Stern 2002). As the hypoblast (and Koller’s sickle) is the main source of FGF signalling at these early stages, this raises the question of whether FGF signalling is required for convergent extension during primitive streak formation. To address this, beads soaked in a FGF signalling inhibitor, SU5402 (Bertocchini et al. 2004; Mohammadi et al. 1997; Streit et al. 2000), were grafted either into the posterior area pellucida of the embryo (to assess the effect on normal development) (Fig. 4A), or into the anterior area pellucida together with a cVG1-expressing cell pellet (to test whether FGF signalling is required for cVG1 to induce a primitive streak and/or the PCP in an ectopic location) (Fig. 4F). SU5402 treatment inhibited *TBXT* expression in the posterior region; it also repressed the induction of *TBXT* by a cVG1 pellet (Fig. 4B, C, G and H), indicating that FGF signalling is required both for maintenance and for induction of *TBXT*. However, SU5402 treatment did not inhibit either the induction or the endogenous expression of *PRICKLE1* in similar experiments (Fig. 4D, E, I and J), suggesting that FGF signalling is not required for *PRICKLE1* expression. FGF signals are transduced through the mitogen‑activated protein kinase pathway via MEK1/2 (Huang et al. 1995; Kouhara et al. 1997). To confirm the results obtained with SU5402, we conducted comparable experiments with a selective inhibitor of MEK1/2, U0126 (using the inactive analogue U0124 as control) (Fig. 5) (Favata et al. 1998). U0126, but not the control, treatment yielded similar results to SU5402, suggesting that MAPK signalling is required for maintenance and induction of *TBXT* but not for *PRICKLE1*. Together, these results suggest that FGF/MAPK signalling is necessary for cVG1 to induce *TBXT*, but not for its induction of *PRICKLE1*.

**Fig. 6:**
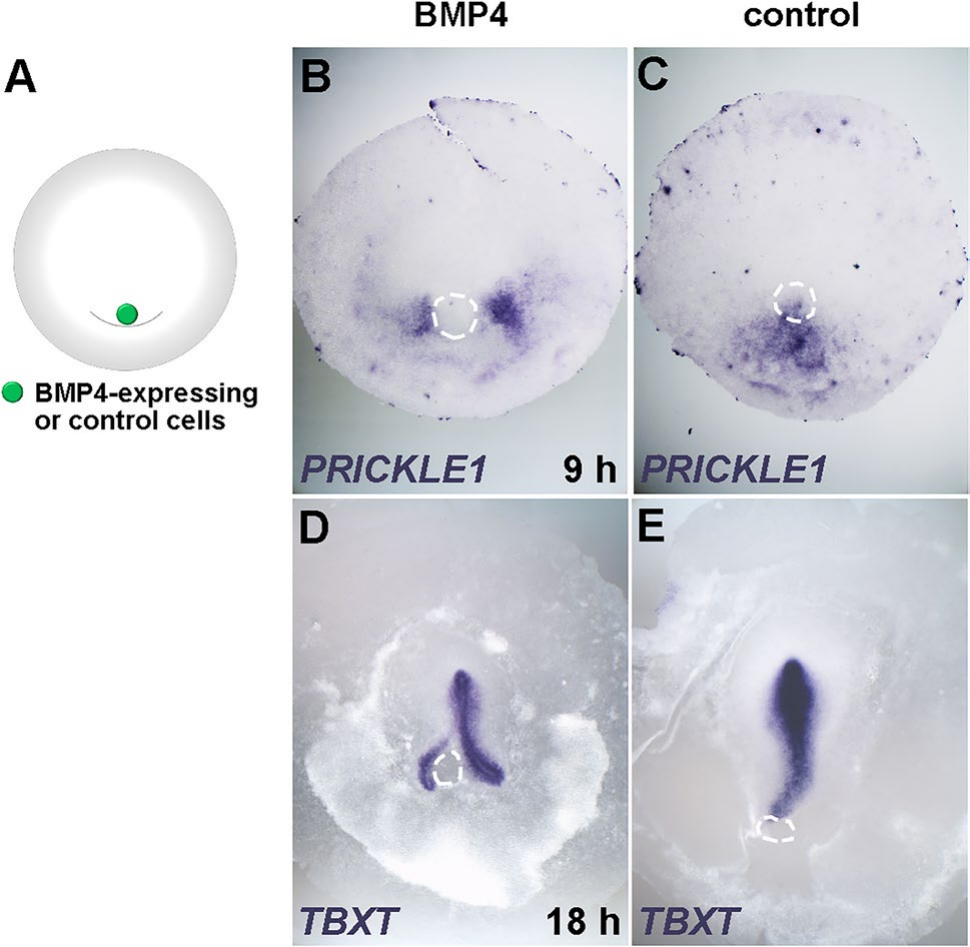
Inhibition of *TBXT* and *PRICKLE1* by BMP4 misexpression. (**A**) Experimental design. A BMP4-expressing cell pellet is grafted to the posterior region of the embryo at EGK X-XI, and the expression of *PRICKLE1* (after 9-h incubation) (**B**–**C**) or *TBXT* (after overnight incubation) (**D**–**E**) was investigated by in situ hybridisation. BMP4 misexpression splits the primitive streak forming region, sometimes generating a double primitive streak, one on either side of the grafted cell pellet (**B**, **D**). Dotted circles, location of the cell pellet. Number of embryos showing inhibitory effect and total number of embryos: 7/7, 0/5, 5/6 and 0/5, respectively, for **B**–**E**

Finally, we tested the effects of misexpression of BMP4, an inhibitor of primitive streak formation that acts antagonistically to cVG1 (Bertocchini and Stern 2012; Streit et al. 1998). Grafts of BMP4-expressing cell pellets onto the posterior area pellucida (Fig. 6A) caused a gap in the domain of expression of *PRICKLE1* (at 9 h) and generated a split primitive streak after overnight culture (Fig. 6B–E). This result is consistent with previous findings that cVG1 is required for primitive streak formation (Bertocchini and Stern 2012), and also suggest that cVG1 may be involved in regulating *PRICKLE1* expression. One interesting possibility is that PRICKLE1 expression is normally repressed by BMP (which exists in the early embryo as a gradient, decreasing posteriorly), and that BMP activity can in turn be inhibited by either FGF (perhaps through phosphorylation of the linker region of Smad1; Demagny et al. 2014; Demagny and De Robertis 2016; Pera et al. 2003) or by Vg1/GDF (perhaps by competition for Smad4; Candia et al. 1997).

## Conclusion

Our results suggest that specific markers for two different cellular events (mesoderm specification and convergent extension) that characterise the cells destined to form the primitive streak are differentially regulated by the upstream signals, cVG1 and FGF8. As the cells in the primitive-streak forming region also undergo EMT and ingression while experiencing other signals (for example WNT, NODAL, BMP etc.), it will be of interest in future to study how those different signals interact to accomplish several cellular events.

## Data Availability

All data contributing to this paper will be made available by the authors upon request, without restrictions.
